# A deliberative study of public attitudes towards sharing genomic data within NHS genomic medicine services in England

**DOI:** 10.1177/0963662520942132

**Published:** 2020-07-15

**Authors:** Lamiece Hassan, Ann Dalton, Carrie Hammond, Mary Patricia Tully

**Affiliations:** The University of Manchester, UK; Sheffield Children’s NHS Foundation Trust, UK; The University of Manchester, UK

**Keywords:** data sharing, ethics, genetic testing, public understanding of science, qualitative research

## Abstract

Whole genome (DNA) sequencing is becoming part of routine care healthcare in England. Genomic data are most useful when pooled with other patients’ data, meaning that clinicians may need to share data to effectively treat patients. We ran deliberative focus groups to explore views among 44 patients and members of the public about proposals for wider genomic data sharing for clinical care. Participants were briefed about genomic medicine and engaged in group and individual exercises to deliberate on the benefits and risks of using genomic data. Findings showed that participants supported wider sharing of genomic data within health services and naturally linked care and research activities. Nonetheless, they were concerned about managing flows of information to protect patient confidentiality and guard against unauthorised uses, now and over the long-term. Ongoing conversations with the public are needed to determine appropriate uses of genomic data and safeguards to inform service development.

## 1. Introduction

Advances in DNA sequencing technologies are revolutionising the delivery of healthcare. Although quick and accurate tests for certain genetic diseases caused by pathogenic variants in single genes (e.g. Huntington disease) have been available to patients since the 1990s (Alford et al., 1994; Harper et al., 2000), rapid scientific advances mean that a person’s complete set of DNA – known as their ‘genome’ – can now be sequenced. The Human Genome Project (HGP), an international 13-year project to sequence the entire human genome, completed its ground breaking work in 2003 at a cost of US$2.7 billion (International Human Genome Sequencing Consortium, 2004; Green et al., 2015; Lander et al., 2001). Since then, the resources needed to sequence the genome have reduced substantially. In 2019, the estimated cost of sequencing an entire genome had reduced to approximately US$1000 (Wetterstrand, n.d.).

Modern genomic sequencing techniques involve comparing an individual’s DNA against a common ‘reference’ genome to identify genetic variants across multiple genes. In economic terms, data of all kinds have been touted as ‘the new oil’ (The Economist, 2017) and genomic data in particular is widely regarded as holding considerable value, both scientifically and commercially (Office for Life Sciences, 2017). Yet, genomic data have greatest utility when pooled and in the context of other linked data about health and phenotypes. Countries such as the United Kingdom and the United States have invested heavily in building and sharing data from so-called ‘biobanks’; large-scale repositories generating genomic data as international resources for research and analysis (Green et al., 2015; Sudlow et al., 2015; Turnbull et al., 2018). These databases are providing a basis for identifying a standard reference genome and increasing our knowledge of genetic variation within and between populations.

### Ethical context: Privacy and consent

Running alongside such scientific and policy developments in genomic medicine has been a rich commentary addressing the ethical, legal and social implications of collecting, using and sharing genomic data (Dheensa et al., 2018; Heeney et al., 2011; Kaye, 2012; Laurie et al., 2010; Shabani and Borry, 2018). Genomic data are both unique to individuals and have a long ‘shelf life’, with test results remaining valid and relevant throughout an individual’s lifetime. Although the notion of whether genomic data should be considered any more sensitive or identifiable than other types of health data has been contested (Davies, 2017), genomic data is still commonly considered highly personal as reflected by descriptions in the popular media as, for example, ‘the ultimate personal information’ (Reynolds, 2017).

Privacy is a fundamental ethical principle in healthcare and research, but when it comes to genomic data, there are ongoing challenges with protecting the anonymity of individuals (Gutmann, 2013; Heeney et al., 2011; Raisaro et al., 2014). For example, Homer et al. (2008) showed that by cross-referencing genomic datasets, even when personal identifiers were removed and data were presented in aggregate, it was still possible to identify an individual in the data. Risks of re-identification may be amplified with the possibility of linkage between existing and emerging data sources, in ways that we may find difficult to anticipate (Heeney et al., 2011). Such observations have prompted certain genomic projects to tighten access restrictions on publicly accessible databases (Church et al., 2009).

Another source of debate has been the extent to which individuals can and should provide informed consent when undergoing genomic testing for different data uses (Laurie et al., 2010). Legal and governance frameworks do differ between providing such data for research and for clinical care. In the context of compiling genomic databases for the former, requiring consent for specific purposes can be restrictive: given the dramatic evolution of genomic medicine in recent years, we cannot know how scientific, technical and policy developments will affect what can be understood and achieved in future. Rather, the standard arrangement recently has been that individuals participate on the basis of broad consent for a range of possible future research uses (Laurie et al., 2010).

The picture is arguably more complex with respect to consenting to sharing genomic data as part of clinical care. As the National Data Guardian in the United Kingdom has acknowledged, to effectively treat one patient, a clinician may need to access data about another who is not under their care (The National Data Guardian, 2016). This is particularly so in genomic medicine. Thus, in some ways, genomic medicine blurs the lines between clinical care and research. While offering flexibility, broad consent models may not be workable in routine care (House of Commons Science and Technology Committee, 2018). Nor may they be strictly necessary under the European Union’s (EU) General Data Protection Regulation (GDPR): according to one interpretation, genetic data collected during the course of care may legally be used for research purposes without consent, providing that data are suitably anonymised (Shabani and Borry, 2018). When it comes to re-using health data, however, prior experience has taught us that legal authority does not necessarily equal social acceptability; to adequately respond to public concerns about the reuse of health data, researchers may need go beyond legal requirements (Carter et al., 2015).

### The evolution of genomic medicine services in the United Kingdom

In the United Kingdom, the government has for some time been making concerted efforts towards mainstreaming genomic technologies within the National Health Service (NHS) (Davies, 2017; Human Genomics Strategy Group, 2012; Office for Life Sciences, 2017). Genetic testing in the NHS is among the best in the world (Human Genomics Strategy Group, 2012). In 2016, the current Chief Medical Officer’s annual report was devoted to outlining her ‘genomic dream’ of further increasing the equitability and availability of genomic medicine services (GMS) (Davies, 2017).

The next phase in implementing this vision is the roll out of the new national ‘Genomic Medicine Service’ for England scheduled for April 2020. This will see the formation of 13 regional centres across the country tasked with delivering a new genomic medicine pathway for NHS patients, a key part of which will involve embedding whole genome sequencing into routine care. These centres will be supported by national infrastructure, including a network of laboratories, a database to aid research and trials and a test directory setting out appropriate tests for particular clinical indications (Robinson, 2020).

Plans for the new GMS build on the successes of the United Kingdom’s 100,000 Genomes Project (100kGP), an ambitious challenge launched in 2012 to sequence 100,000 whole genomes and in doing so, make the United Kingdom a world leader in genomic medicine (Turnbull et al., 2018). The project has been regarded as a vanguard not only in terms of its scale but also in respect of its hybrid nature – for the first time in the NHS, patients were required to agree to participate in ongoing research to obtain genomic testing (Human Genomics Strategy Group, 2012). Having reached its stated target of sequencing 100,000 genomes in December 2018, the success of 100kGP has helped to make the case for wider genomic testing in the NHS (Robinson, 2020).

### Towards a new social contract

With whole genome sequencing set to move into routine NHS services, there has been increasing discussion about how these new arrangements could affect the nature of the existing ‘social contract’ between patients, the public and staff that is currently enshrined in the NHS constitution; a document setting out the rights and responsibilities for NHS patients, staff and the public (Bukowski et al., 2019; Davies, 2017; Dheensa et al., 2018).

Although there are well-established ethical debates on sharing genomic data altruistically and solely for research purposes, sharing genomic data more freely within the NHS carries additional implications for privacy, consent processes and models of information governance. Unlike submitting genomic data to a repository to be used solely for research, for example, under the arrangements of the new GMS, individual patients and their families might personally stand to benefit from undergoing sequencing themselves and/or via others agreeing to provide samples. Thus, service developments may conceivably result in altered perceptions of the risks and benefits, and rights and responsibilities, for each party.

There is widespread agreement on the importance of understanding and engaging with public views if such progressive visions for collecting, sharing and using health and genomic data as part of future care are to be realised (Garland, 1999; Human Genomics Strategy Group, 2012; Shabani et al., 2014; The Nuffield Council on Bioethics, 2015). While projects such as 100kGP have set a precedent for hybrid projects and have at least partially addressed such issues (Dheensa et al., 2018), uses of genomic data solely for clinical care arguably remain a distinct issue. There have been calls for further investigations and national ongoing conversations in this area (Davies, 2017; Shabani et al., 2014).

In light of plans to mainstream genomic data within NHS services, the aim of this study was to understand public values, interests and concerns in relation to sharing genomic data within the NHS for the specific purpose of clinical care. The purpose of this was to influence the future development of the GMS in line with public values and inform efforts to inform and engage patients and the wider public about appropriate uses of genomic data.

## 2. Method

### Design and setting

The study was conceived by AD in response to local discussions about establishing regional GMS in Northern England and then designed by all authors. We ran six qualitative, deliberative focus groups to explore opinions about genomic data sharing in the course of routine care within the NHS. Ethical approval was granted by a research ethics committee run by the Health Research Authority (ref: 18/NW/0510).

A qualitative approach was chosen as this is suited to exploring views on complex areas. In addition, deliberative approaches allow for in-person provision of unbiased information about relevant underpinning concepts, as presentations and answers to questions, which is useful in situations where members of the public may have limited pre-existing knowledge on which to base their views.

### Recruitment and sampling

We used a purposive sampling strategy to recruit from two key populations: (a) three groups for patients and family members who had experienced contact with genetic services; and (b) three groups for members of the general public with no experience of genetic services and no particular interest in the area (Table 1). We set a maximum of 10 places per focus group. Two groups were reserved specifically for young people; one for patients and one for the public, both held at weekends. Young people aged 18 years could choose to attend groups designated either for young people or adults. Groups were held in Manchester and Sheffield, both of which are large, ethnically diverse cities in Northern England.

**Table 1.** Focus group types and locations.

Group	Type	Age (years)	Location	Number of attendees
1	Adult public	18+	Manchester	10
2	Young public	16–18	Manchester	9
3	Adult public	18+	Sheffield	9
4	Adult patient	18+	Sheffield	7
5	Young patient	16–18	Sheffield	4
6	Adult patient	18+	Sheffield	5

Advertisements were circulated via online research recruitment platforms, patient and public involvement networks, university and high school email newsletters, physical noticeboards (including libraries and supermarkets) and social media (including Facebook and Twitter). For the purposes of recruiting people with experience of existing genetic services and their carers, a genetic counsellor (CH) approached patients of the Sheffield Genetics Service, either in person or via post, with study invitations enclosed within routine correspondence.

In the three focus groups held for members of public, where there were far more applicants than places (over 150 applicants), participants were selected using quota sampling techniques to reasonably mirror local populations in terms of gender, age and ethnicity. For the patient focus groups, where there were many fewer applicants, we accepted all applicants who met the inclusion criteria and were available to attend. Participants were excluded if they were aged under 16 years; did not speak or understand verbal English; worked as part of the NHS as a healthcare professional; and/or had learning disabilities or difficulties that would preclude their participation.

All participants received an information sheet and gave informed consent to participate. A payment of £100 (provided as cash for adults and high street vouchers for young people) was provided as reimbursement for participation.

### Deliberative focus group design

An agenda and topic guides were prepared to structure the day and guide discussions (see Supplemental material A–B). Three hypothetical scenarios about fictional characters (Fred, Mary and Sally) were developed by the team to stimulate discussion about how genomic data might be used in the future GMS (see Supplemental material C). One scenario was further refined for clarity following the first focus group.

All materials were reviewed and edited in response to comments by a patient and public involvement group associated with the Sheffield Genetics Service. The agenda, topic guide, scenarios and slides shown to participants were also reviewed for bias and clarity by two independent panellists (and revised where necessary). Both were separate from the research team and funders, and had relevant expertise in genomics, governance of health data, deliberative methods and public engagement.

### Conduct of focus groups

Deliberative focus groups were carried out between September 2018 and January 2019 following the approach described by Rothwell et al. (2016). Each group was moderated by two facilitators. The first was an experienced meeting facilitator, independent of the research team, who led discussions and directed the group work throughout the day, keeping the participants on track. The second was a subject expert (AD), an expert in genomic data use, who gave a 30-minute presentation on genomic medicine (see Supplemental material D), services and data use followed by a question and answer session. She remained throughout the day to answer questions of fact (but not give opinions) during the group discussions. A third team member (LH or MT) attended to take informed written consent, record notes and ensure consistency of data collection procedures. For ethical reasons, the genetic counsellor attended focus groups involving patients to answer queries, provide support and signpost services if required. Young people were invited to bring parents or carers to accompany them if they wished, though they were not permitted to participate in discussions.

On arrival, participants completed a short questionnaire to provide demographic information and, where applicable, basic details about their experience of existing genetic services. After introductory exercises to establish their level of background knowledge, participants were briefed about genomic medicine by the subject expert. The remainder of the day involved a combination of group discussions, practical exercises (e.g. ranking reasons for and against data sharing) and individual deliberation about the benefits and risks of using genomic data.

### Analysis

Following the focus group, recordings were transcribed verbatim by a professional transcription service. Transcripts were then coded and analysed by two team members (LH and MT) using NVivo software (version 12) and a thematic approach (Braun and Clarke, 2006). Briefly, transcripts were initially coded according to a pre-determined framework of descriptive codes based around the topics addressed in the topic guide. Researchers worked on separate transcripts initially and then met periodically to review each other’s coding, revise the coding framework accordingly and exchange ideas about the developing analysis. Original recordings were revisited where necessary to ensure accuracy of interpretation. Thematic categories were merged, refined and adapted until a final set of themes and suitably illustrative exemplar quotes were selected. Ellipses [. . .] indicate where superfluous text has been removed.

## 3. Results

Overall, 44 participants took part (Table 2), with slightly more women than men. Participants who attended focus groups for the general public were more ethnically diverse than those in groups for patients.

**Table 2.** Sample characteristics for patient and public attendees.

	Participant counts (n)		
	Patients	Public	All
**Gender**			
Male	4	14	18
Female	12	14	26
**Ethnicity**			
White	16	18	33
Black and minority ethnic groups	0	11	11
**Age (years)**			
16–17	3	8	11
18–24	1	6	7
25–34	1	3	4
35–44	4	6	10
45–54	2	2	4
55–64	3	2	5
65–74	2	1	3
75+	3	8	11
**Employment status**			
Employed	7	11	18
Retired	2	2	4
Student	3	8	11
Unemployed	2	5	7
Other	2	2	4
**Highest level of education**			
School	6	8	14
Vocational	3	2	5
A-level	1	5	6
Degree or above	6	13	19
**Total**	16	28	44

The results that follow refer to the sample as a whole, unless otherwise indicated. Where views were held by particular subgroups of participants, these will be drawn out in the text.

### Pre-existing knowledge/reference points

The strongest themes from opening discussions about what participants already knew as to the meaning of genes, genomes and testing were those of heritability and individuality:

What makes us unique. (Focus Group 6, adult patients)

You inherit them from your parents. (Focus Group 5, young patients)

I just feel like it’s what makes you different. (Focus Group 5, young patients)

The most common reference point for genetic testing was screening for genetically related conditions (personal, familial and in pregnancy) and informing treatment decisions related to any such diagnosis. Participants also mentioned uses of genetic testing related to establishing paternity, ancestry and forensic science.

Knowledge and perspectives about genetic testing appeared to be grounded in formal education, but also popular media, including reality TV shows ( ‘that programme where celebrities track their heritage’), factual science pieces and science fiction.

We did not find substantial differences between patients and members of the public in terms of their general level of background knowledge of genetics. Some individuals – mainly, but not always, patients – were well-informed about the genetic aspects of specific conditions from personal or familial experience; however, this did not necessarily translate into broader general knowledge:

I put chromosome disorders, because that’s what I kind of know about, and then not so rare chromosome disorders like Down’s syndrome, and then some types of cancer. Lots of things, but don’t really know what. (Focus Group 4, adult patients)

Even when discussing conditions relevant to themselves or their relations, participants’ frequently expressed uncertainties in their knowledge. Several participants actively sought reassurance and clarification from genetic experts on hand during later discussions regarding aspects of heritability, prognosis and treatment.

### Supporting diagnosis and treatment for individual patients and their families

Both patients and the wider public supported the sharing of genomic data within the NHS to support the diagnosis and treatment of people with genetic conditions and their families. Participants viewed collecting and storing genomic data over the long-term about individual patients presenting for treatment as a necessary – even obvious – step to allow diagnoses and informing decisions about their treatments, now and in the future. This was acceptable, but under the proviso that each patient who was seen by the service had full knowledge of how data about them would be used and had expressly given their permission:

Obviously we’ve got benefits – the treatment, he [Fred] gets the treatment for the condition, therapy, access to help like that . . . There’s a benefit for his family, as [name] pointed out. A man of that age might have a wife and family, dependants, financial responsibilities, so being diagnosed early on, being potentially treated for that condition would be a benefit for that situation. (Focus Group 4, adult patients)

Instead of treating everybody all on one level you can actually target and give them specific individual treatment which will give them the best chance of surviving. (Focus Group 5, young patients)

For some participants, the logical conclusion of creating a database would be that genomic data among family members would eventually be linked. Younger people saw clear benefits to having data on parents and other family members to help understand familial risk factors and inform their own life choices:

Obviously, there’s loads of benefits about sharing his data [Fred], his care, for his children, their children, to be able to identify treatment that works, any effects that it might have later on his life as well. (Focus Group 6, adult patients)

If he [Fred] had a record that his father had died of motor neurone disease then he would just have to wait for a blood test that comes back saying you’ve got it and we’d be able to start treatment straightaway. So it kind of carries over that his offspring should be aware. (Focus Group 2, young public)

Patients in particular commented on how sharing data between different health professionals and specialisms – particularly where cases were more complex or involved several co-existing conditions – could provide additional context and reduce reliance on patients to repeatedly provide the same information to different health professionals. This could help to make care for individual patients more joined up, ‘holistic’ and efficient:

You do tend to find that people tend to be stuck within their own speciality and don’t tend to look at other aspects of the person as a whole. (Focus Group 4, adult patients)

I think just one of the small benefits is not having to repeat yourself constantly every time you change hospitals. (Focus Group 5, young patients)

### Treating others and the ‘greater good’

There was widespread support for using data as a collective resource for the GMS to draw upon when diagnosing and treating patients, other than themselves. The scenarios provided described how the genomic data of patients with similar conditions or genetic profiles could be compared to aid diagnosis and treatment.

Participants recognised that even if there was no immediate direct benefit to a particular patient or their family by contributing their genomic data to a shared database, pooling genomic data could be used to benefit other patients, now and in the future. This was conceptualised as benefitting others or contributing to the ‘greater good’:

Yeah, so if you share, it’ll improve diagnosis and treatment. And if you don’t, it can slow it all down. (Focus Group 1, adult public)

There isn’t any kind of correlation that can be found that can give her [Mary] any treatment directly herself. So the main benefit in sharing her information would be to increase the amount of knowledge in the genetic database which would theoretically help other people in the long run, researchers, other people with similar conditions to her . . . (Focus Group 4, adult patents)

While participants felt that they should be informed about how their data would be used and asked to give their permission, the consensus was that sharing within the NHS, in the manner depicted in the scenarios, should become ‘the norm’. Given the choice, it was believed that few would exercise their right to opt out, although some admitted they had not always held this opinion:

I think that should just become the system that is the norm. That this is what we do with data. Everybody who’s a member . . . is using the NHS should know that that’s what’ll happen. (Focus Group 1, adult public)

Sometimes we felt that our views change. Right at the very beginning we didn’t have a good understanding of what difference it could make to the future, and so you get bombarded with data so you just tick, tick, tick, no, no, no, but you don’t really understand all that. Six months down the line you realise wow, sharing this information is going to make a real big difference for the future. (Focus Group 4, adult public)

### Data security

A consistent theme raised by participants across all groups concerned security and unauthorised access to data stored by the GMS. Overall, these issues ranked most highly among the lists of potential risks raised by participants.

There was general agreement that the sensitivity of data warranted attention to data security. However, there were differences among participants in terms of where (and whom) they perceived threats to come from. Some participants had general concerns about data security, recognising the vulnerability of any cloud-based IT system to unauthorised users, those acting with malicious intent (e.g. ‘hackers’), misuse and accidental losses.

Several participants raised more specific concerns about their impressions of the NHS’s track record with IT and data security, citing examples of incidents that had been reported in the media including cyber attacks on the NHS, patient data being lost and failed IT initiatives:

We’ve actually had incidences of patient data being lost, being left on trains, on USB sticks, on CD-ROMs that can just end up in the public domain like that. So, you can say that it’s secure, but nothing is 100 per cent secure, we know that. (Focus Group 4, adult public)

If this was me having motor neurone disease, I’d be happy for like the NHS because they’re the ones making me better but then again, the whole cloud thing like you hear stories about the NHS getting hacked and everything. (Focus Group 2, young public)

Although there was an acceptance that genomic data needed to be stored securely, some participants – particularly younger people – did not always comprehend how their genomic data in particular could be valuable to others who did not personally know them:

Hackers and misuses, it’s a worry but ultimately, you know, like for me, I don’t really know what information they’re going to find that they find interesting, you know. (Focus Group 5, young patient)

I don’t understand why of all the things you’d hack, you’d hack genome sequencing . . . I mean, if it’s purely genes, I don’t understand, like if it was hacked and the information was leaked to a third party, I don’t understand what would be the use of that information. (Focus Group 2, young public)

Where younger people did show specific concerns, this tended to be oriented towards protecting information against unauthorised disclosures to third parties such as parents, future or current employers and law enforcement services. Adult participants were also concerned with limiting access by third parties, including commercial companies, such as marketing companies and insurers:

I’d like a guarantee that it was only going to be circulated to health professionals . . . That it’s not going to be used for marketing purposes and won’t be sold on, that it’s not a profit thing. (Focus Group 3, adult public)

### Confidentiality and managing ‘need to know’ information

In the first and simplest scenario (Fred), the general consensus was that the patient presenting for treatment should be ‘told everything’ upfront; not only any results, but also how the data would be stored, shared, used in future and by whom. However, as the scenarios became more complex, we observed careful deliberation among participants regarding the wider implications of full disclosure and whether this was realistic, and indeed desirable.

The third scenario presented raised the possibility of previously unknown familial connections being unearthed as a result of investigating disease outcomes in individuals on the database (Mary and Sally) with strong genetic similarities. In this case, providing the fullest information possible to an individual patient was balanced against the need to preserve wider confidentiality and avoid unintended disclosures:

But should the service actually be allowed to let people know that they have relations, relatives they didn’t know existed? (Focus Group 4, adult patient)

As discussions about what should be communicated progressed, the emphasis moved from ‘full disclosure’ to talk of a ‘need to know’ basis, as the following exchange demonstrates:

1: And what should Mary be told about what is being shared? I don’t really think she needs to know that her information is being shared.

2: To help others, that’s what I just wrote.

1: Yes, because in the cloud it is used to help others, so she doesn’t need to know the logistics of what’s happening

2: You get told what you need to know, don’t you, and that’s it.

1: You don’t get, ‘oh, so you want to know about Percy who’s just been here last, he had the exact same condition as you’. (Focus group 1, Adult public)

Several safeguards were identified and discussed by groups. A commonly expressed caveat was that data shared outside of the patient’s immediate care team would need to be depersonalised in some manner to protect confidentiality. Some participants also expressed preferences for differing levels of access among different types of services and healthcare staff:

What we were saying was there should be two clouds, primary and secondary, so the primary one should be for your GP, so the GP knows everything, your name, your age, all that stuff, and you have a secondary one which should be hospitals and stuff, as in the genes. (Focus Group 2, young public)

As discussions progressed, participants increasingly recognised the implications of wider data sharing and that changes in how services intended to share and use genomic data were not trivial. Thus, there needed to be serious consideration about how information would be managed and communicated to patients:

The risk here is that you’re going to uncover relationships, family relationships that weren’t known about, that didn’t exist, they could be there for a number of reasons, it could be adoption, it could be cases of adultery; and how people respond to that knowledge, I don’t think you can necessarily predict that by ticking a few boxes. (Focus Group 4, adult patient)

### ‘Once it’s out there’: Wider uses, mission creep and uncertain futures

While research was not explicitly mentioned within the scenarios, patients in particular were quick to point out that sharing data might build an evidence base and enable research to understand genetic conditions and develop new treatments for people affected by these conditions and their families:

Also one benefit of sharing the data is that you’re building up knowledge; and the rarer the condition the more important it is to share the data. (Focus Group 4, adult patient)

With a lot of them, they sort of come down to help save lives, you know, so building all the research helps saves lives. Helping to raise awareness and provide accurate diagnosis for family members is helping save lives, you know. (Focus Group 6)

In some focus groups, there were unprompted discussions about the risks of how data might be used in unforeseen ways in the near or distant future. Even if satisfactory terms of use and safeguards were arranged at the current time, some people – mainly older adults – recognised the commercial value of data and expressed concerns that changes in government, legislation or policy might have an impact on future approved uses of any genomic database that might be created:

At the moment we are looking at a future where everything is supposedly going to be locked down . . . So, with the right pressure to the government, big companies come in and say well we’d like access to this kind data it would help us to do x, y and z. People get backhanders, nice holidays abroad, next think you know legislation is passed. Hey ho – your information is being used by big corporations for their own purposes. (Focus Group 3, adult patient)

It’s not a trivial task, in my opinion, to set out these safeguards, it’s complicated and it involves ethical areas as well. It really does need to be taken very seriously to make sure that what we have here isn’t going to be used for nefarious or evil purposes in the future, or isn’t going to become something that causes further problems down the line. (Focus Group 4, adult patient)

In this context, these future intended uses were conceptualised as potentially legal, yet unethical and/or undesirable uses and were thus separate from outright illegal activities or accidental data breaches. Fears expressed included the impact of potential privatisation of the NHS, data being released to commercial companies, data being used for genetic selection purposes and data being shared with the police for forensic purposes. Thus, the focus group participants were aware that decisions made about access to and use of data may need to be revisited regularly as the context of the NHS and data use more generally changed.

## 4. Discussion and conclusion

In exploring the views of both the general public and patients, our study meaningfully contributes towards the growing body of knowledge that addresses public opinions about sharing genomic data within the context of routine healthcare (Bukowski et al., 2019; Shabani et al., 2014).

After deliberating on the intended purposes and scenarios presented, we found there was support for creating a collective resource of genomic data that could be built up over time and drawn upon more widely within regional NHS GMS. The strongest reasons for supporting collection and sharing of data in this way were improving the speed and accuracy of diagnosis and providing more personalised care to individuals, their families and other patients using the service. The focus on improved patient outcomes in exchange for wider data sharing is highly consistent with previous research that has found the public are most supportive of uses of genomic and wider health data that will deliver tangible benefits to patients (Aitken et al., 2016; Lemke et al., 2010; Tully et al., 2018).

While deliberately excluded from our example scenarios, participants spontaneously recognised the potential of wider data sharing, outside of the GMS, to further contribute towards the ‘greater good’, supporting greater knowledge through research into genetic conditions. This indicates that in the context of genomic medicine, care and research are connected in the minds of patients and the general public. For some people, increasing interplay between these two practices is a predictable and natural direction of travel in genomic medicine, with data sharing forming part of the wider trade-off around accessing and improving NHS services. The attitudes we observed in this study may well have been influenced by the continued high level of attention that genetic medicine discoveries receive in the popular media (Bubela and Caulfield, 2004), as well as the significant public engagement and recruitment campaigns undertaken to support major UK biobanking projects (Samuel and Farsides, 2018).

The main risks associated with collecting and using genomic data were perceived to be around protecting privacy and managing flows of information. Participants expected that precautions would be taken to secure their data and restrict access to appropriate people for appropriate purposes; however, they had subtly different views on who and what these were. From a psychometric perspective, risks may mean different things to different people (Slovic et al., 1982). We found that young people were especially concerned with managing their privacy and potential disclosures of information among parties who might be interested in their health status, including parents and prospective employers. In an era where it is commonplace for young people to choose to disclose details of their private lives online, this concern with privacy may seem surprising to some. But, risks associated with data sharing activities may be perceived as more acceptable (and manageable) if entered into voluntarily (Starr et al., 1976). In this sense our findings are consistent with other studies that have found that younger people place importance on access controls and privacy from defined groups in relation to their health data (The Royal Academy of Engineering, 2010; Trinidad et al., 2010).

We did, however, note that concerns about the potential longer term risks of creating genomic resources – including commercial access and genetic discrimination – were largely absent from discussions among younger people. In a previous UK study, younger people did show concerns about commercial access to health data (The Royal Academy of Engineering, 2010). It is possible that by focusing our materials solely on uses of data within regional NHS GMS, possible concerns about commercial access remained undisclosed. The work of Laurie et al. (2010) on dimensions of privacy may also be relevant here. Younger people appear to place particular value on what these researchers termed ‘decisional privacy’, exercising control and choice over who has access to their data. It could be, however, that their sense of ‘informational privacy’ – that is, awareness of the implications for misuse and discrimination through uses of data, whether anonymised or not – may be less well developed.

Our findings reiterate the need for considering the timing and delivery of information to patients about data sharing alongside any changes to the existing genetic services. This could have implications for training healthcare professionals to strike the right balance between informing patents and overwhelming them and/or paternalism (Bester et al., 2016; Ha et al., 2018). We also recognise that new developments fundamentally affect the NHS’ ‘social contract’ and thus support wider calls for broader, more critical public conversations about acceptable policies for governing uses of genomic data (Bukowski et al., 2019; Davies, 2017; Dheensa et al., 2018; Samuel and Farsides, 2018). Given ongoing concerns expressed about ‘mission creep’ and commercial access to health data, these should occur irrespective of technical measures to gather permissions, protect anonymity or secure data.

In addressing public opinions about sharing genomic data, in many ways, our study addresses familiar territory. However, retaining a strict focus on usage within routine healthcare arguably sets this study apart. By using deliberative focus groups, we were able to provide participants with information, as well as opportunities for reflection, group discussion and deliberation. This allowed a greater level of engagement with the topic than could have been achieved by surveys or standard focus groups. Even over the course of just 1 day, we observed shifts in participants’ opinions.

While we took steps to minimise bias, inevitably our choices of materials and speakers will have influenced participant responses. For example, we included only one expert speaker; though intended to be impartial, as an NHS employee, it is possible that participants perceived her perspective differently. We did not include other speakers to present opposing views (e.g. privacy experts and data sharing advocates), as have other deliberative studies of health data (Tully et al., 2018). Nor did we explicitly address the topic of public–private partnerships within the NHS or the prospect of future commercial involvement in the context of GMS, which is a known concern for some (Dheensa et al., 2018). Thus, while this topic did spontaneously occur in some groups, we were unable to elucidate all discussants’ perspectives on this matter. We acknowledge that these design choices, imposed partly to retain focus within available resources, may have neglected the finer details of infrastructure underpinning the GMS and implicitly encouraged participants to support data sharing.

The sample size was in keeping with other qualitative studies of this type (Aitken et al., 2016; Lemke et al., 2010). Groups for the general public were well balanced with respect to age, gender and ethnicity. We deliberately sought out and over-sampled young people in recognition that this demographic group are often not included in such work but have most opportunity to be affected by developments in the GMS throughout their lives. Patient groups were smaller and lacked diversity. In particular, we lacked representation from Black and other minority ethnic groups, who are known to both be under-represented in genomics research and have distinct views on data sharing (Bukowski et al., 2019; Haga and O’Daniel, 2011). This is a limitation of the study and should be addressed by future studies.

Further integration of genomic medicine into care carries implications for engagement with existing, previous and potential service users. As with previous studies (Bukowski et al., 2019), we found that the background level of ‘genomic literacy’ among many participants was relatively low; furthermore, knowledge did not substantially differ between members of the public and patients. Participants in our study generally welcomed greater access to information and influence; however, they recognised that this could not be a one-off conversation, and nor could full disclosure or control be feasible in all situations.

## Supplemental Material

Supplemental material for A deliberative study of public attitudes towards sharing genomic data within NHS genomic medicine services in England Click here for additional data file. Supplemental material, Supplemental_Material for A deliberative study of public attitudes towards sharing genomic data within NHS genomic medicine services in England by Lamiece Hassan, Ann Dalton, Carrie Hammond and Mary Patricia Tully in Public Understanding of Science
