# Supplementary material for: A deliberative study of public attitudes towards sharing genomic data within NHS genomic medicine services in England
Source: Public Underst Sci. 2020 Jul 15;29(7):702–17. doi: 10.1177/0963662520942132 (PMC7539600; doi:10.1177/0963662520942132)

A deliberative study of public attitudes towards sharing genomic data within NHS genomic medicine services in England

DOI:10.1177/0963662520942132

Dr Lamiece Hassan (corresponding author)

Centre for Health Informatics, Division of Informatics, Imaging and Data Sciences, School of Health Sciences, The University of Manchester, Manchester, Oxford Road, M13 9PL, UK. Email: lamiece.hassan@manchester.ac.uk

Prof Ann Dalton

Sheffield Diagnostic Genetics Service, Sheffield Children's NHS Foundation Trust, Western Bank, Sheffield S10 2TH. Email: anndalton2015@gmail.com

Dr Carrie Hammond

Sheffield Diagnostic Genetics Service, Sheffield Children's NHS Foundation Trust, Western Bank, Sheffield S10 2TH. Email: carrie.hammond@nhs.net

Dr Mary Patricia Tully

Division of Pharmacy and Optometry, School of Health Sciences, Faculty of Biology, Medicine and Health, University of Manchester, Manchester Academic Health Science Centre, Manchester, UK. mary.p.tully@manchester.ac.uk

Table of contents - supplementary files

File A – Agenda..2

File B – Topic guides..3

File C - Scenarios..6

File D - Expert presentation briefing and slides..8

File A - Agenda

Sharing your genome in the NHS: a deliberative study of public opinion on data sharing within genomic medicine services

Time (duration)	Activity and purpose	Document references
9:30 (30 mins)	Coffee, registration, consent check and questionnaire	PIS and consent form
10:00 (15 mins)	Housekeeping, ground rules, aims and introductions	
10:15 (20 mins)	Discussion 1: Background knowledge, experience and perceptions	Topic guide – discussion 1a/b (File B)
10:35 (40 mins)	Expert briefing: Educational presentation from expert co-moderator, question and answer session and post-presentation discussion<br>*Purpose:* To bring everyone up to speed by providing information on how regional Genomic Medicine Services (GMS) work, including how data is shared in order to provide care. To explore views on the GMS. To understand individual perspectives and responses to the information presented.	Expert briefing guide (File D) & Topic guide - discussion 2 (File B)
11:15 (15 mins)	Break and regroup.	
11:30 (30 mins)	Discussion 2: Views on data sharing in the GMS. To understand individual perspectives and responses to the information presented.<br>*Purpose:* To explore knowledge, experience and perspectives on data sharing as part of services.	Topic guide – discussion 3 (File B)
12:00 (45 mins)	Scenario discussion and feedback in groups<br>*Purpose:* Discussion of scenarios involving genomic data. Feedback and questions.	Scenario guide (File C)
12:45 (45 mins)	Lunch	
13:30 (60 mins)	Scenario discussion and feedback in groups<br>*Purpose:* Discussion of scenarios involving genomic data. Feedback and questions.	Scenario guide (File C)
14:30 (15 mins)	Break and regroup.	
14:45 (30 mins)	Discussion 2: Informing patients and the public<br>*Purpose:* To understand what patients and the public would want to know about the GMS and how this information should be communicated.	Topic guide – discussion 4 (File B)
15: 15 (30 mins)	Summarise key points, final remarks, debrief.	
15:45 (15 mins)	Evaluation and payments	
16:00	Close	

File B - Topic guides

Discussion 1a (people with no experience of services)

Tell me everything that comes to mind when I say the term 'genetics/genes'? What does that mean to you? Write down your ideas and first impressions on the post it notes (*one thought per post it*).

1. How could your genes impact on your health?
   - What are the sorts of things that come to mind?
   - What types of conditions or health problems might be linked with genetics?
   - What kinds of care, treatment and/or other services might people with these conditions need?

2. In what situations might someone need to take genetic tests or visit a specialist genetics service?
3. Where have you heard/learned about this? *Probe for seeing relevant discussions in news, media, films, school.*

Discussion 1b (people with experience of services)

1. Thinking back to before your first visit, what information were you given about how the service would use data about you?
2. Did you discuss any of this information with your family or others?

Discussion 2

Pre-presentation introduction

Next we will hear from an expert in genetic medicine who will explain a little about how 'genomic medicine services' work. Take a moment to think about what kinds of questions you have about this topic. As they are talking please write any questions that you have down on the post it notes provided and we can answer them at the end. Don't worry if you forget any at this stage – the expert will be around all day and you can always ask more questions later.

Post-presentation discussion guide with focus group participants

Q&A to follow presentation – use post-it notes to prompt discussion.

1. Did you learn anything?
2. Were there any parts of the service that were unexpected or surprising to you?
3. Did anything contradict what you thought you knew? (*Refer back to earlier discussion*)
4. Imagine a doctor had referred you to this service OR think back to when you first used one of these services. What else might you want to know/have wanted to know about how a genomic medicine service works?

Discussion 3

Introduce the idea that we are going to focus on how data stored in patient medical records is used and shared within genomic medicine services for the care of each individual patient. Explain the role of the expert facilitator in this discussion before beginning. Record and group ideas on flipcharts and post it notes.

WHAT/WHY?

1. What kinds of 'data' or information do you think genomic medicine services might collect and use in the course of diagnosing and treating patients? Tell me everything that comes to mind. *Probe for examples*
    - What could it be used for?
    - How sensitive is it?
    - Could it be linked to you and/or your family?
    - Is it identifiable, coded in some way and/or anonymous?
    - Would it be linked to/part of your other health records (e.g. GP, hospital)?

Ask expert for input/comment – Q&A

WHO/WHY?

2. Who do you think might record information in the medical records of patients' visiting the genomic medicine service?
    - What kinds of information would they record?

3. Can you think of anyone else who might need to see/use information in medical records within the genomic medicine service? Why might they need to see/use it? *Explore different people, departments and potential uses.*
    - How comfortable are you with each of these types of people/groups having access to this data?
    - Who are you least/most comfortable with having access?
    - Would you be expect to be asked for permission to share your data/records?

Ask expert for input/comment – Q&A

HOW?

4. What safeguards or regulations do you think are in place around how patient data and medical records are used within genomic medicine services?
    - What would reassure you?
    - What would concern you?

Ask expert for input/comment – Q&A

SUMMING UP

Ask for the key points and/or most important things that we've learned this morning and want to bear in mind – then print these clearly on flipcharts and display these on the walls for the rest of the day.

Discussion 4

Explain that now we want to understand a little about what patients and the public would want to know about the new GMS, how data might be used as part of this and how this information should be communicated.

1. Based on the information that you have heard today, what do you think patients and people who use the GMS services would want to know about how their data is used?
    - What information should be provided?
    - How should this be provided? At what point? By whom?

2. In particular, what information (if any) should patients be told about how their data is used as part of the following scenarios?
    - When data about you is shared with others during the course of your own treatment
    - When data about you is shared with others during the course of the treatment of others

3. What information do you think should be available for the wider public (including carers and families) about how genetic medicine services are run?

File C - Scenarios

Scenario 1

- At the age of 47, Fred is diagnosed with motor neurone disease, an uncommon disease that affects the brain and nerves. He recalls that his father died of "something similar" in his early 30s.
- Fred is referred to the new genomic medicine service and agrees to have all of his DNA data 'read' as part of his clinical care. This is called 'genome sequencing'. This reveals a change (mutation) in one of Fred's genes, which has been linked to motor neurone disease. This helps confirm his diagnosis and makes targeted treatment possible.
- Fred's sequencing information and other data about him that was sent as part of his referral are stored electronically by the genomic medicine services in a secure area known as 'the cloud' where Fred's care team can access it. Over time, Fred's information is updated regularly to build a picture about what treatment Fred is given and how he responds.
- Fred's information can be made accessible to other healthcare professionals within the genomics medicine service more widely, who may wish to look at Fred's information to help them diagnose and treat their own patients, including relatives of Fred.

Scenario 2

- It was back in 2009, when Mary was 50 years old, that she was first diagnosed with breast cancer. This led to her having her breast surgically removed.
- Both Mary's mother and aunt died from cancer. Both lived in Nigeria, where Mary was originally from and Mary has not seen her Dad for several years. So it is difficult to get any information regarding the cancer in her relatives or any sample for genetic testing.
- As part of Mary's treatment she had genetic tests, which showed a change in one of her genes. Mary's doctors noted that a case like this was once seen in Singapore, where there was a patient with a similar change who developed a cancer at 60 years old. However, no further details were available and so it was difficult to draw any firm conclusions about the relevance to Mary's case and we cannot offer targeted treatment.
- As part of the new genomics service, all of the data about Mary so far – including her genetic testing results, family history, ethnic origin, pathology results, treatment and outcomes – is put into 'the cloud' for storage.
- Mary's information is made accessible to other healthcare professionals within the genomics medicine service more widely, who may wish to look at information about her case to help them diagnose and treat their own patients.

Scenario 3

- In 2019 Mary's cancer returns. Mary contacts the genomic medicine service as she is worried about whether her sister and daughter may also be at risk.
- More genes linked to cancer have been identified so Mary is offered further genetic testing to screen a wider range of genes associated with cancer. Although the test comes back negative

apart from the previous change seen, the lab noted unusual variations that they had to look at very carefully before ruling them out as being associated with cancer.
- The genomics service adds these results to Mary's other information already held in the cloud. Information about her ongoing care, treatment and outcomes are also added.
- In 2020, a second patient, Sally, presents with breast cancer at age 34. Sally is of mixed heritage and was adopted so we have no information about her parents. Sally agrees to genetic testing. Her doctors review her results against those of other patients in the cloud and upon doing so, find that Sally shows some genetic similarities with Mary, which suggests they may be closely related. If we can link Sally to Mary, we may be able to give more information to Mary's sister and daughter.

Scenario discussion guide

Run through the scenario, ensuring the following information is clear:

- WHAT data is accessed?
- HOW is the data accessed?
- WHO has access?

Split the room into smaller groups. Instruct each group to generate as many ideas as they can for the following:

- What are the possible benefits of sharing data in this way? What might happen if we don't do this?
- What are the possible risks of sharing data in this way? What might happen if we do this?
- What should patients be told about how data about them is shared and used? When should they be told? Who should tell them?
- What else came up? Has any of the information you've heard changed your view at all?

*Ask each table to feedback and post the key reasons on flipcharts around the room. Ask each person to walk around the room and vote for the top 3 benefits (and * the single most important one) and the top 3 risks (and * the single most important one).*

Also ask them to vote on the level of consent required for each scenario to vote on the following:

- Consent implied as part of consenting to NHS treatment- no additional consent required
- One-off overarching consent for the use of my data for this type of activity
- Explicit consent for each such use of my data – even if that happened quite frequently
- Not happy with my data being used for this purpose

File D:
Expert presentation briefing and slides

Briefing guide for the genomics expert facilitator

The expert facilitator will prepare a 15-20 minute presentation for the focus group participants. The aim of this should be to provide information on how Genomic Medicine Services (GMS) work in the region, including how data is shared in order to provide care. You may use videos and/or images as part of your talk, should you wish.

The presentation should include the following information:

- A brief introduction about yourself and your role.
- What is genetic data? Introduction to DNA, genes and the genome
- Understand why and how understanding genomic and molecular data can be used to care for patients.
- Description of the different types of health conditions among people who may come into contact with services and typical patient pathways.
- Description about the way the service is delivered, including the types of services offered (e.g. common tests) and locations where these are delivered.
- Description about the key staff roles in the care team.
- How patient data flows as part of this service currently
- Please note that your slides will be reviewed for bias, completeness and accuracy of content by an independent panel, separate to the project steering group.

GeNEQ

CONNECTED HEALTH CITIES
A nhsa project

NHS

Sharing your Genome in the NHS

Professor Ann Dalton

Director Sheffield Diagnostic Genetics Service

Sheffield Children’s NHS Foundation Trust

Inherited Disease – diagnosis, prognosis, treatment

Prenatal Diagnosis

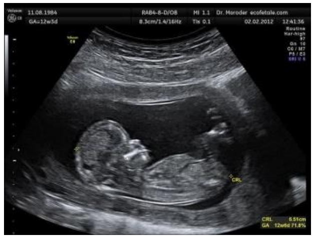

Newborn Screening

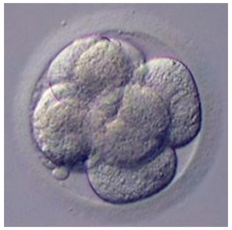

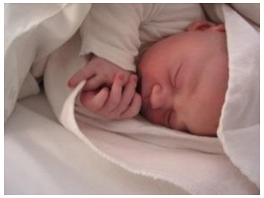

Pharmacogenomics

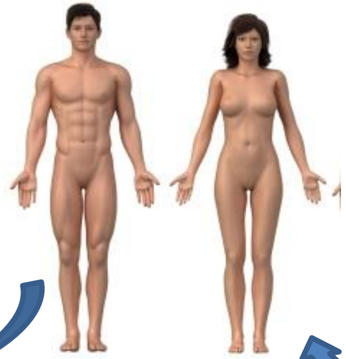

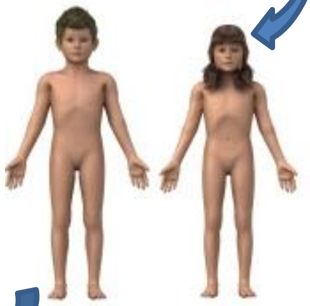

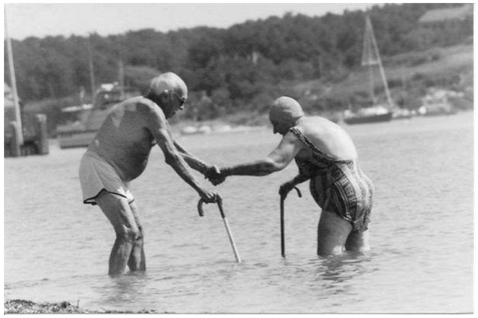

Cancer – diagnosis, prognosis, treatment

DeoxyriboNucleic Acid – the instruction booklet

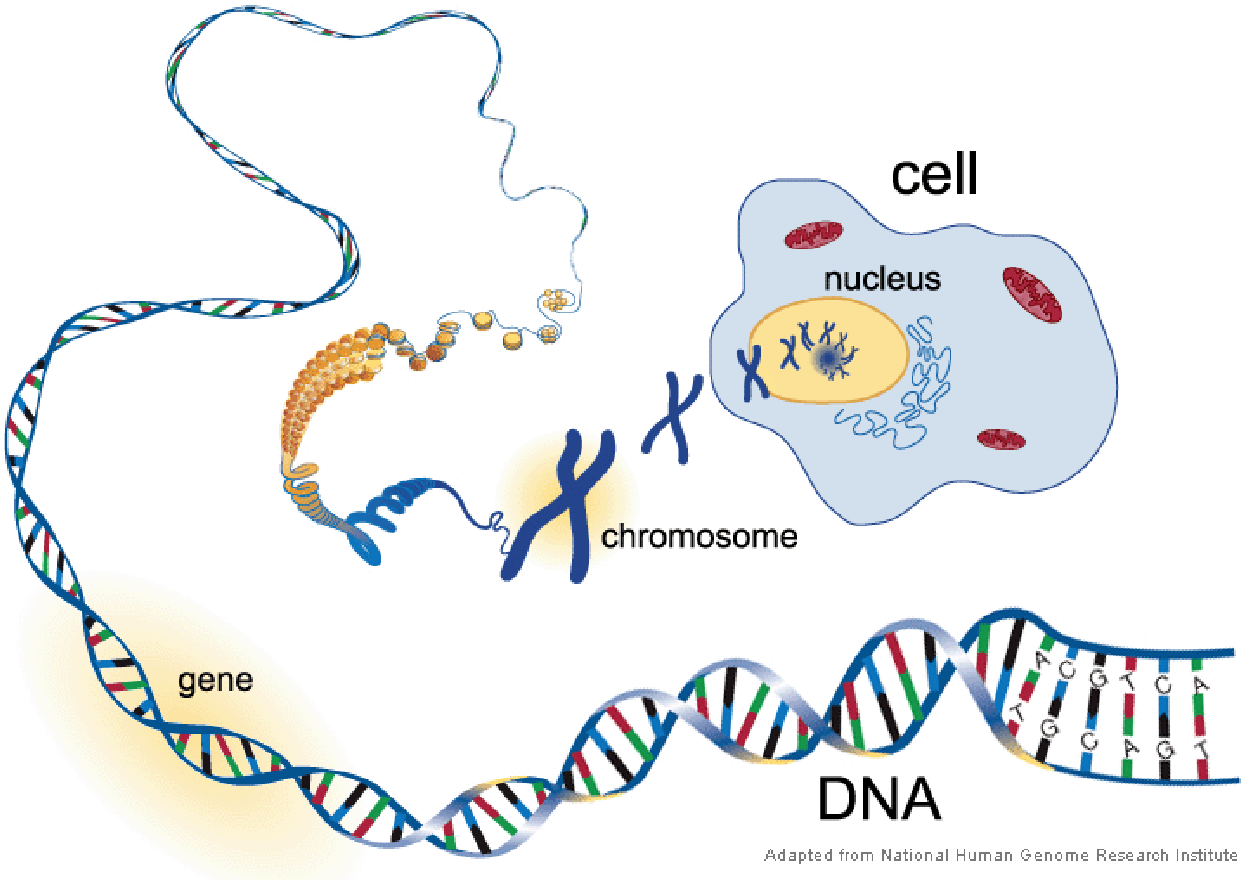

Mistakes/glitches

The cat sat on the mat

The cat sat in the mat

The cat sat on the hat

The cat spat on the mat

Variants of uncertain significance

- Whole Genome sequencing – 3 billion base pairs – 5 million variants

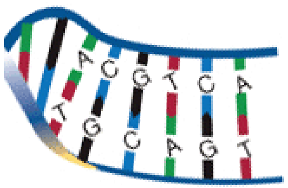

- In genes only 25-30,000
- Exclude common polymorphisms – 500

cat

- Check inheritance pattern 50-100

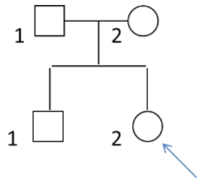

- Candidate mutations – 2-3

How do we know what is important?

- Literature and research
- Databases
- Understanding the underlying chemistry and associated biology
- Other members of the family
- Other people with the same variant who are not related

GeNEQ

Genomic Medicine Service

Equitable genomic services

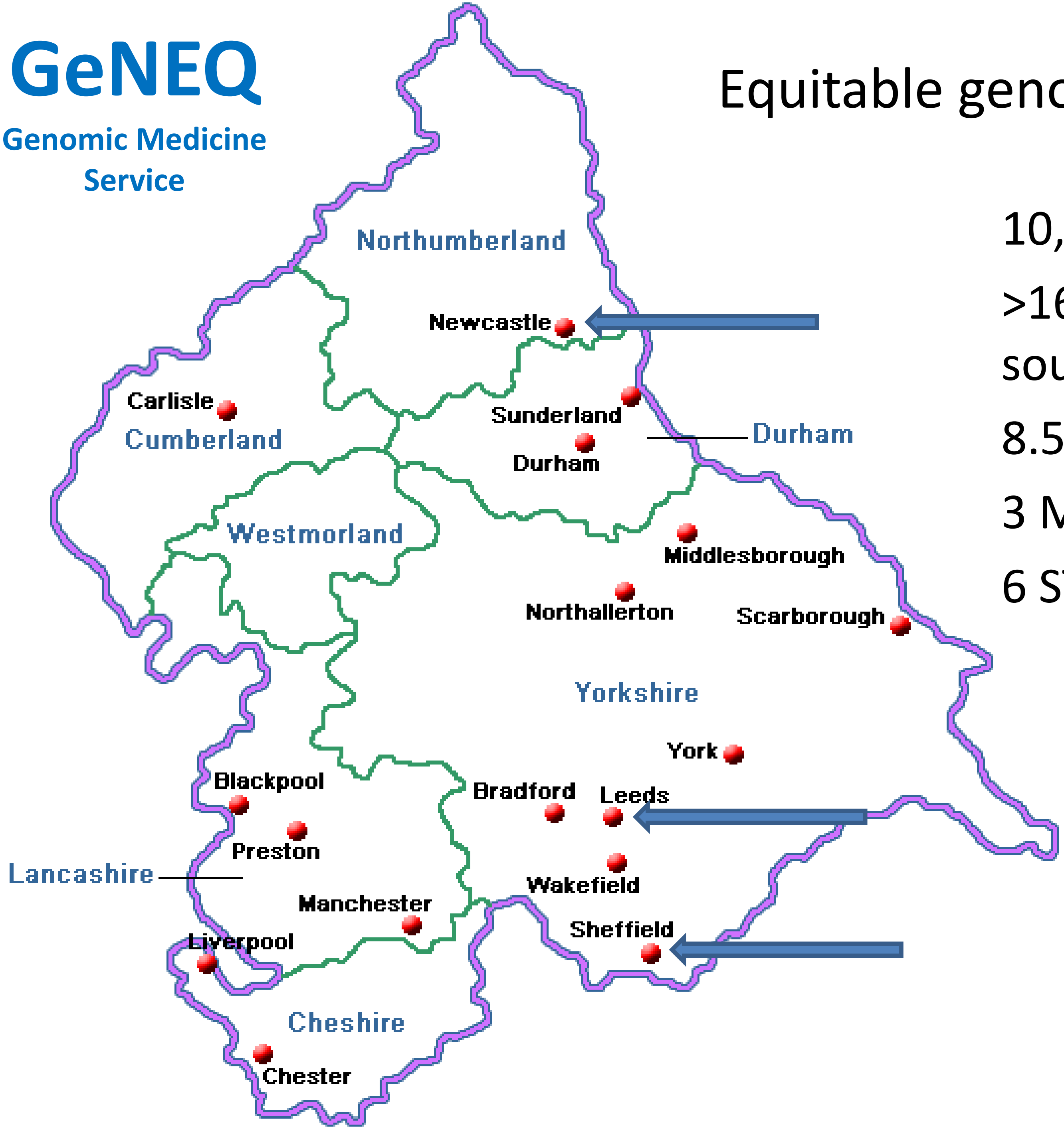

- 10,000 sq miles
- >165 miles north-south and east-west
- 8.5 million people
- 3 Major hubs
- 6 STPs

What does a Genomic Medicine Service do?

- Sees patients with inherited or acquired disease
- Tests the (right person and) right tissue at the right time to reach a diagnosis, inform prognosis and guide treatment
- Informs the patients and their families
- Works with other healthcare professionals in all branches of medicine to provide integrated, high quality, equitable care.

Why we need to share data?

- To understand and interpret the genomic results for you, your family and people who have a similar condition to you
- The more we share the data, the more likely it is that we can give the correct advice and treatment and potentially the more 'personalised' that information can be

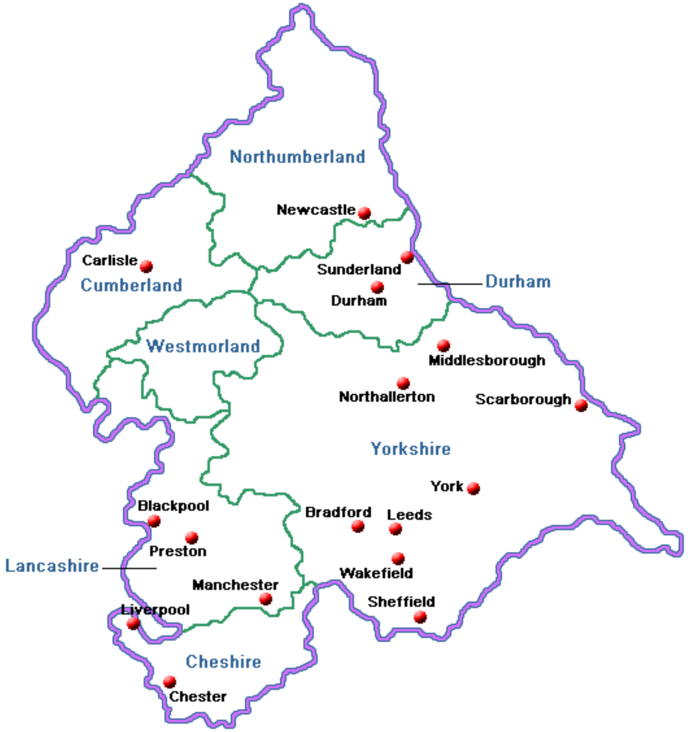

Familiarity, Identifiability and Longevity

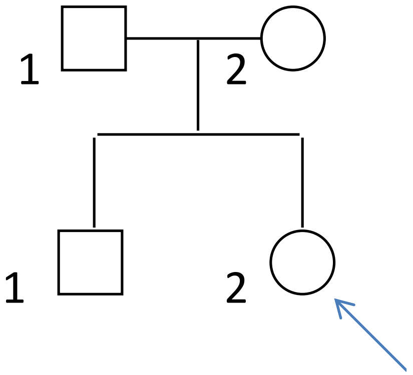

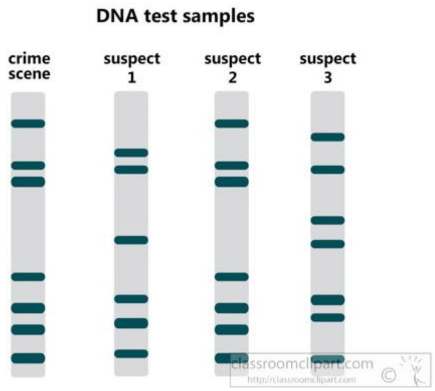

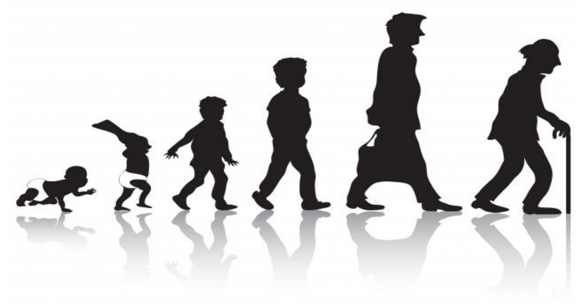

Who will look at your data?

- Doctors
- Scientists
- Nurses
- Counsellors
- Administrators

How does it differ from what we do now?

- We will be required to share (some) data with the national database for genomics (which is being built)
- We want to share data (genomic data and the patient record) freely amongst GeNEQ (which will make GeNEQ work effectively and efficiently)

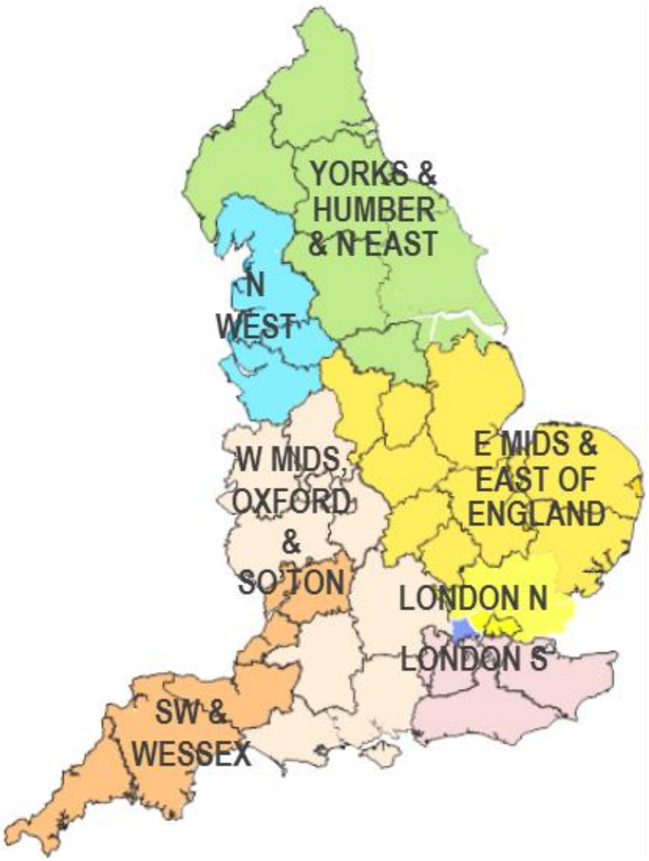

Supplement: Supplemental material for A deliberative study of public attitudes towards sharing genomic data within NHS genomic medicine services in England [file Supplemental_Material.pdf]
